# Supplementary material for: Manipulating the Subcellular Localization and Anticancer Effects of Benzophenothiaziniums by Minor Alterations of N-Alkylation
Source: Molecules. 2023 Feb 10;28(4):1714. doi: 10.3390/molecules28041714 (PMC9965410; doi:10.3390/molecules28041714)
Supplement: Supplementary file 1 [file molecules-28-01714-s001.zip › molecules-2205765-supplementary.pdf]

Supporting information

Manipulating the Subcellular Localization and Anti-cancer Effect of Benzophenothiaziniums by Minor Altertion of N-Alkylation

Yanping Wu,[a] Yuncong Chen,[a,b*] Shankun Yao,[a] Shumeng Li,[a] Hao Yuan,[a] Fen Qi,[a] Weijiang He,[a*] Zijian Guo[a,b*]

[a] State Key Laboratory of Coordination Chemistry, Coordination Chemistry Institute, School of Chemistry and Chemical Engineering, Nanjing University, Nanjing 210093, PR China

[b] Chemistry and Biomedicine Innovation Center, Nanjing University, Nanjing 210023, PR China

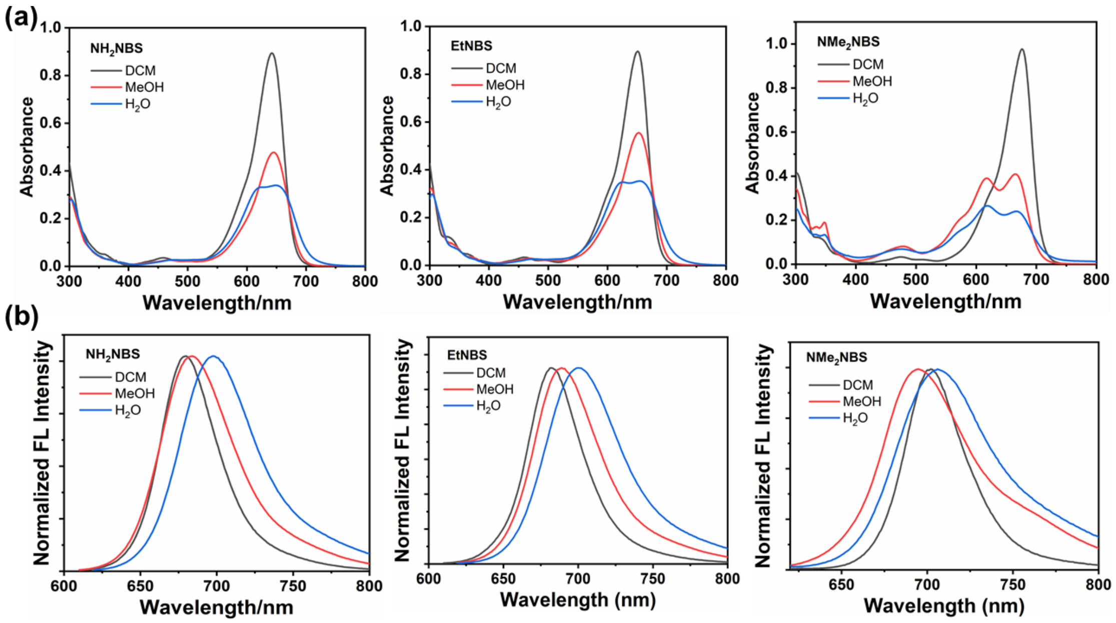

Figure S1. Normalized absorption and emission spectraof NH_2NBS, EtNBS, NMe_2NBS in PBS solvents. The concentration used for each compound was 10 μM.

Table S1. Physical and photophysical properties.

Solvent	Dye	λ_{abs} (nm)	ε($M^{-1}cm^{-1}$)	λ_{em} (nm)	Φ_{fl}	Φ_{Δ}
DCM	NH_2NBS	642	89351	680	0.24	N.D.
	EtNBS	651	89585	682	0.40	N.D.
	NMe_2NBS	676	97661	702	0.20	N.D.
MeOH	NH_2NBS	645	47781	684	0.10	0.057
	EtNBS	653	55495	689	0.14	0.060
	NMe_2NBS	665	40942	695	0.04	0.034
H_2O	NH_2NBS	649	33957	698	0.02	N.D.
	EtNBS	654	35314	700	0.03	N.D.
	NMe_2NBS	667	24061	706	0.015	N.D.

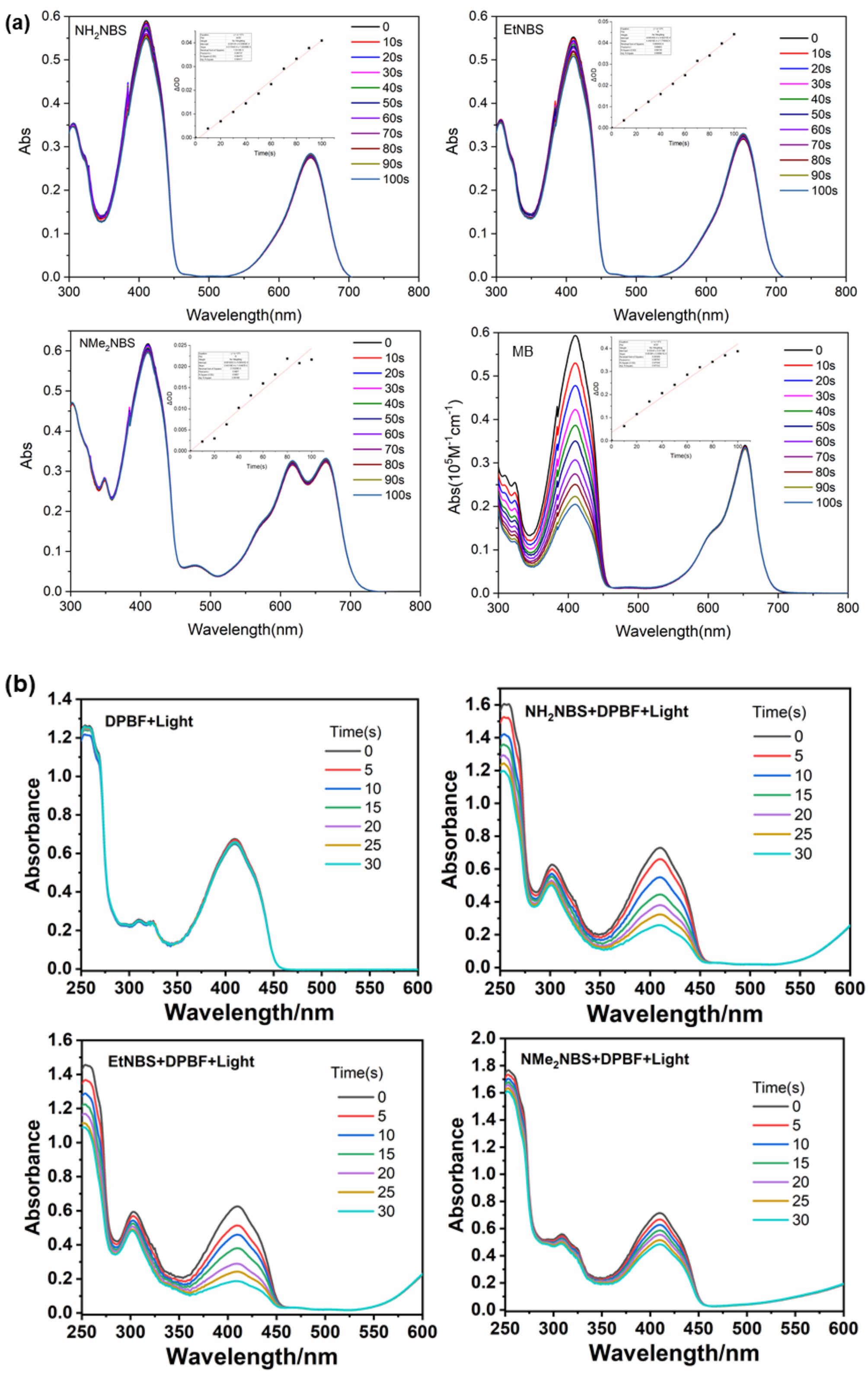

(a)
NH2NBS
EtNBS
NMe2NBS
MB
Abs
Abs(10^5M^{-1}cm^{-1})
Wavelength(nm)
ΔOD
Time(s)
0
10s
20s
30s
40s
50s
60s
70s
80s
90s
100s
(b)
DPBF+Light
NH2NBS+DPBF+Light
EtNBS+DPBF+Light
NMe2NBS+DPBF+Light
Absorbance
Wavelength/nm
Time(s)
0
5
10
15
20
25
30

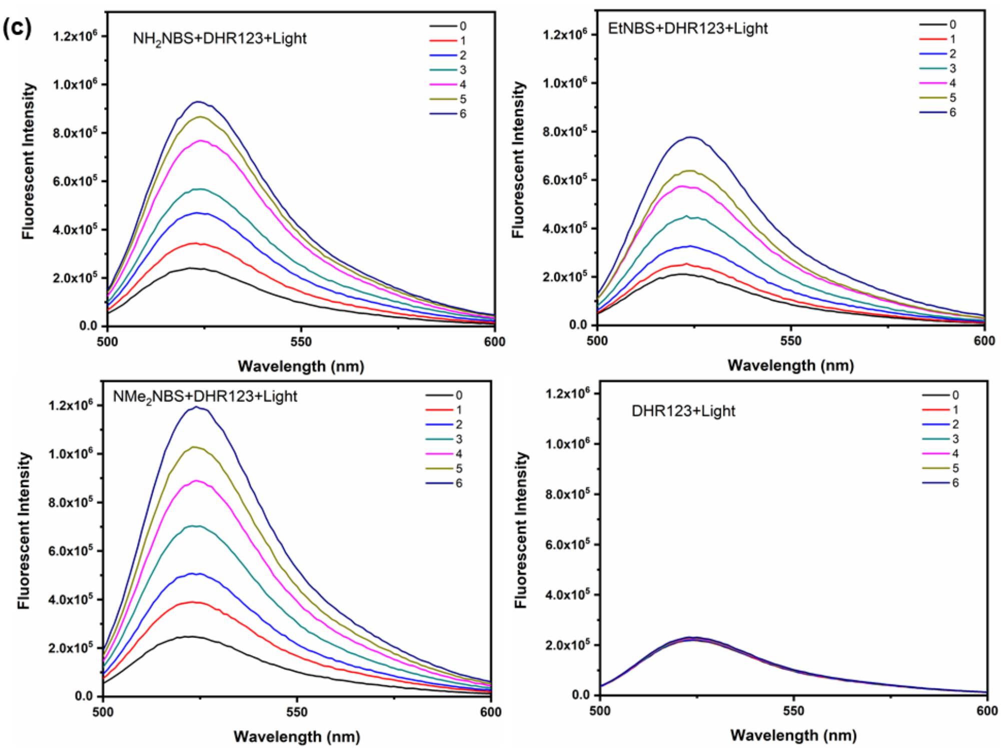

Figure S2. (a) The singlet oxygen quantum yields of the three PSs were determined in MeOH using DPBF bleaching assay (after 635 nm irradiation for 100 seconds, Dye concentration: 10 μM). (b) Photoinduced $^1O_2$ production was determined in MeOH (after 635 nm, 20 mW cm^{-1}, irradiation for 30s, Dye concentration: 10 μM) (c) Photoinduced $O_2^{-\bullet}$ radical generation was determined in PBS solution using DHR 123 (after 635 nm, 20 mW cm^{-1}, irradiation for 6 min, Dye concentration: 10 μM).

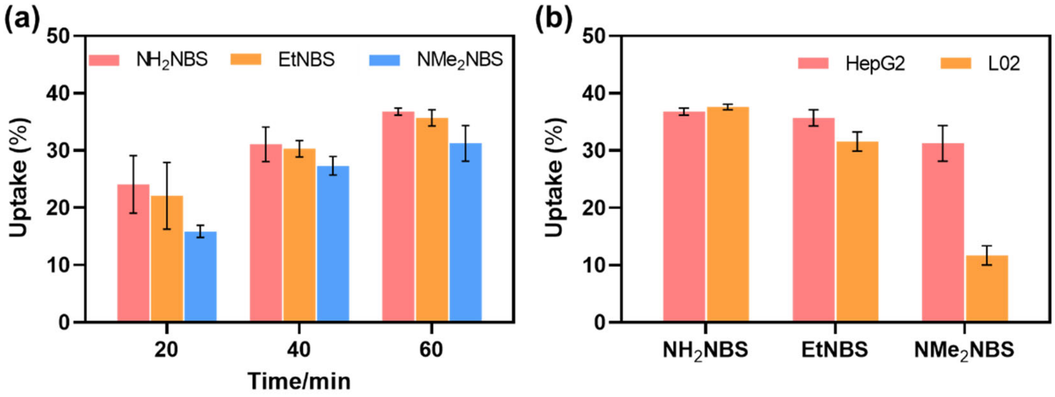

Figure S3. (a) Uptake of three PSs by HepG2 cells at different time intervals. (b) Uptake of three PSs by HepG2 cells and L02 cells after incubation for one hour.

Table. S2 Phototoxicity and dark toxicity of NH_2NBS, EtNBS, NMe_2NBS against L02 cells.

Compound	IC_{50}(μM)		
	Dark	Light (N[a])	PI[c]
**NH_2NBS**	3.00 ± 0.40	0.039 ±0.001	77
**EtNBS**	4.69 ±0.27	0.050 ±0.010	94
**NMe_2NBS**	57.04 ±2.86	1.15 ±0.35	50

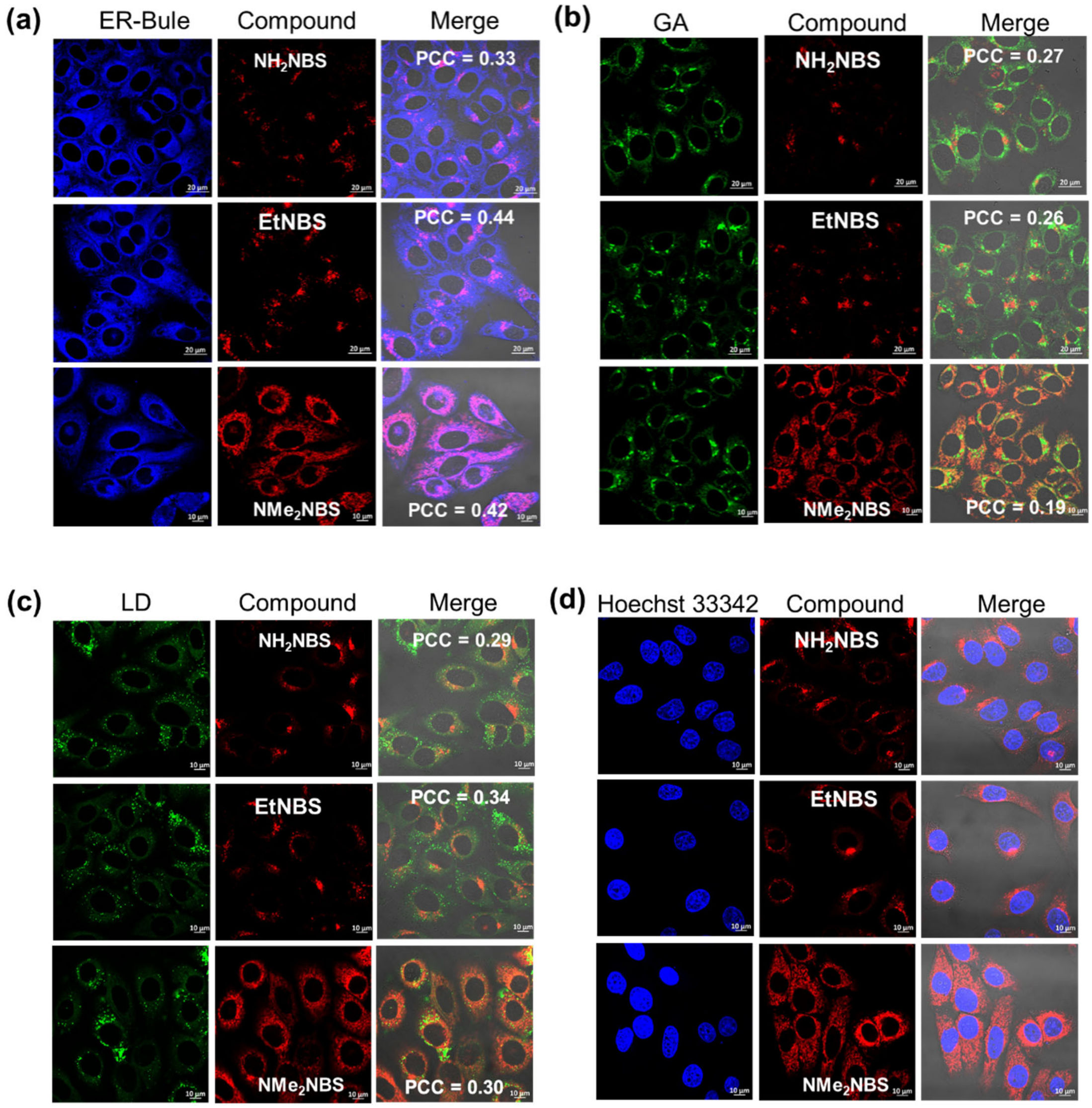

Figure S4. Subcellular localization of NH_2NBS (100 nM), EtNBS (100 nM), NMe_2NBS (100 nM), ER Blue white (1 μM), GA: Golgi apparatus Green (1 μM), LD: Lipid green (1 μM).

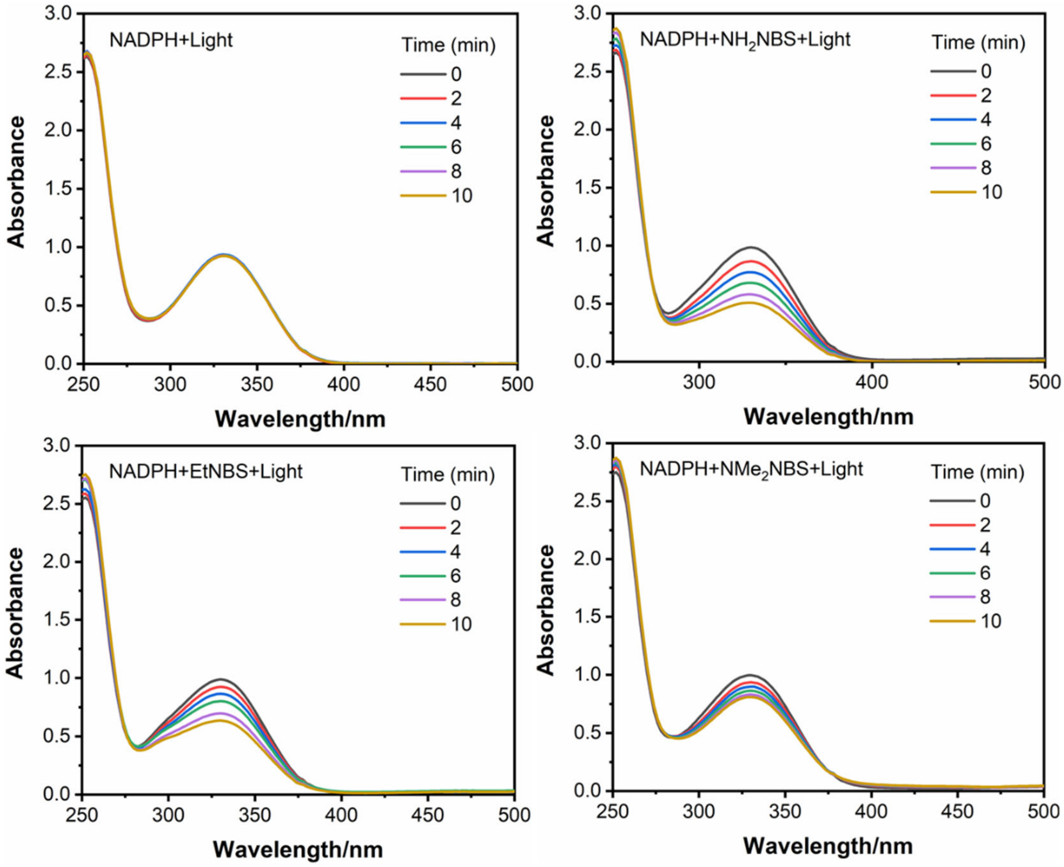

Figure S5. UV-Vis spectra of photocatalytic oxidation of three PSs (10 μM) with NADPH（160 μM) in PBS solution, after different time of 635 nm irradiation at a power density 20 mW/cm^2.

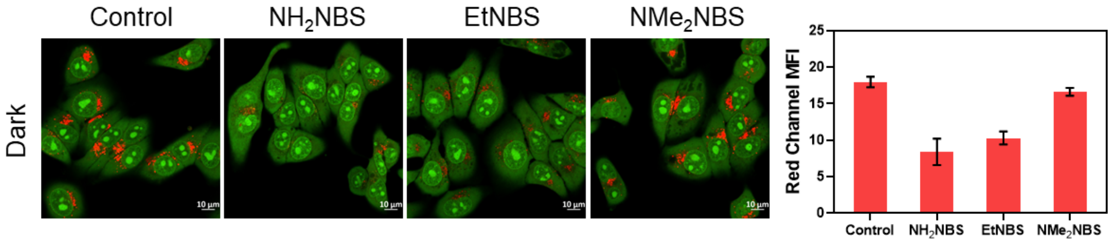

Figure S6. The confocal fluorescence images of AO staining after co-cultured HepG2 cells with high concentration of NH_2NBS, EtNBS and NMe_2NBS (1 μM).

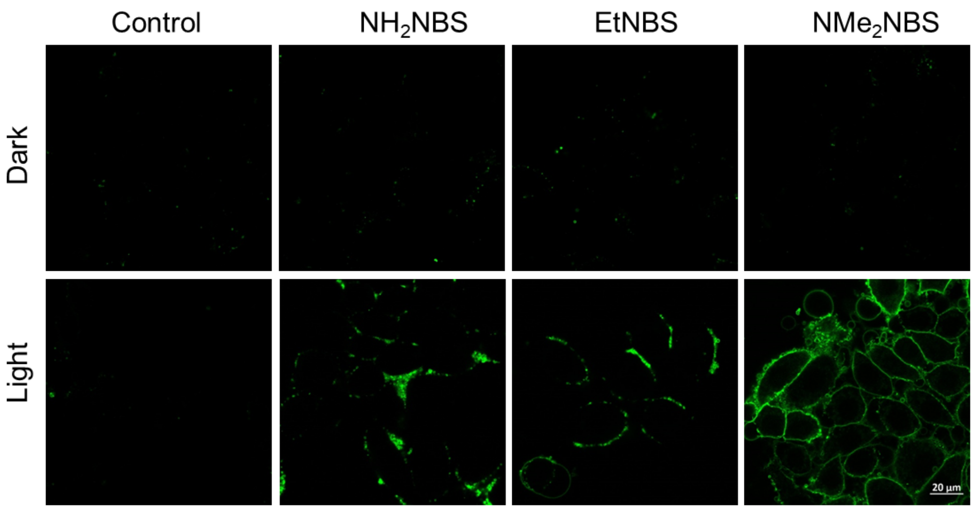

Figure S7. The confocal fluorescence images of Annexin V-FITC staining.

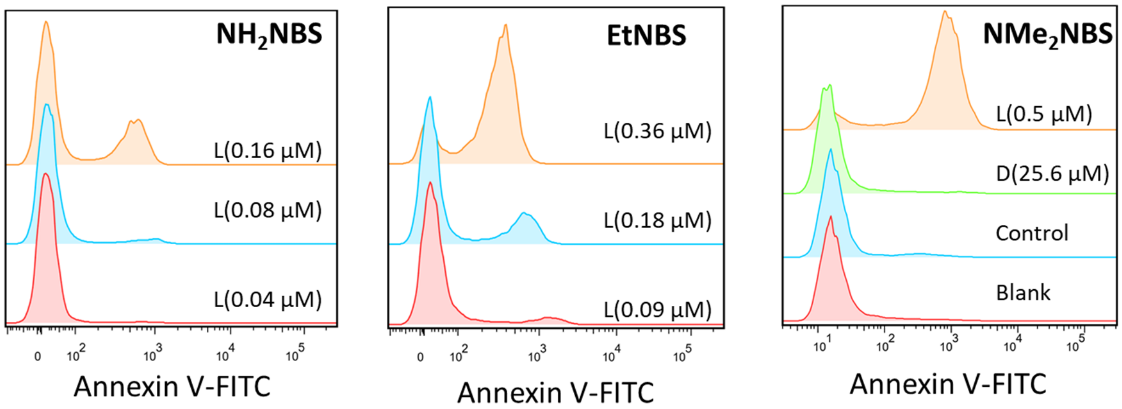

Figure. S8. Flow cytometric assay of photoirradiation-induced apoptosis for HepG2 cells incubated with different concentrations of NH_2NBS, EtNBS and NMe_2NBS. L: light, D: Dark.

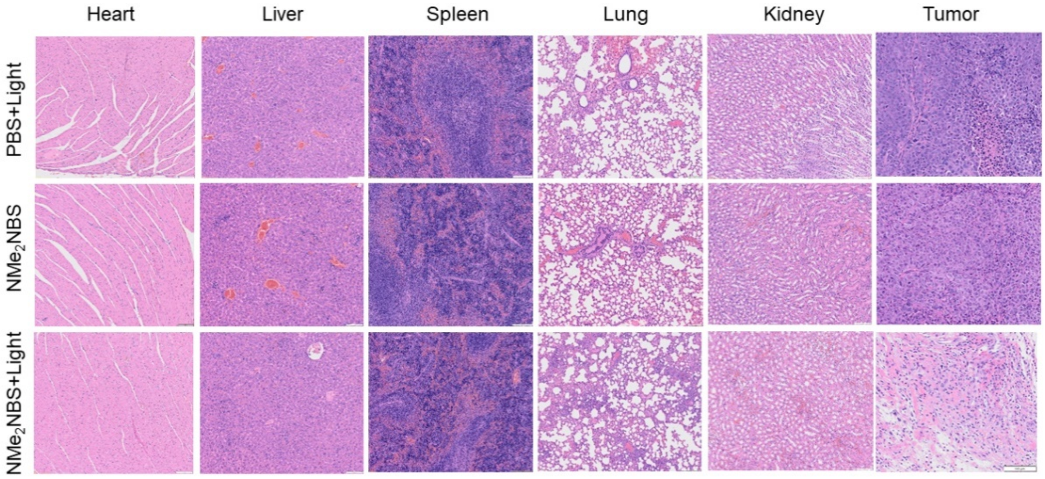

Figure S9. H&E staining of organ tissues and tumor tissue slices after four weeks treatment.

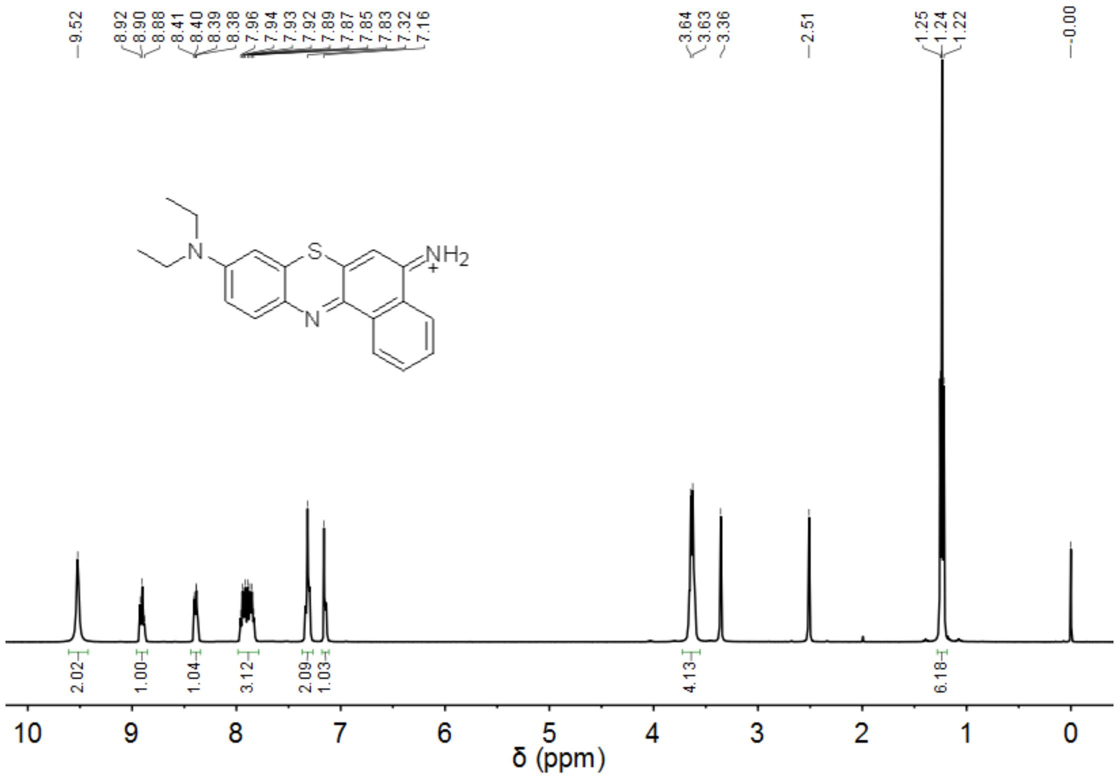

Figure S10. ^{1}HNMR of NH_2NBS in DMSO-d_6.

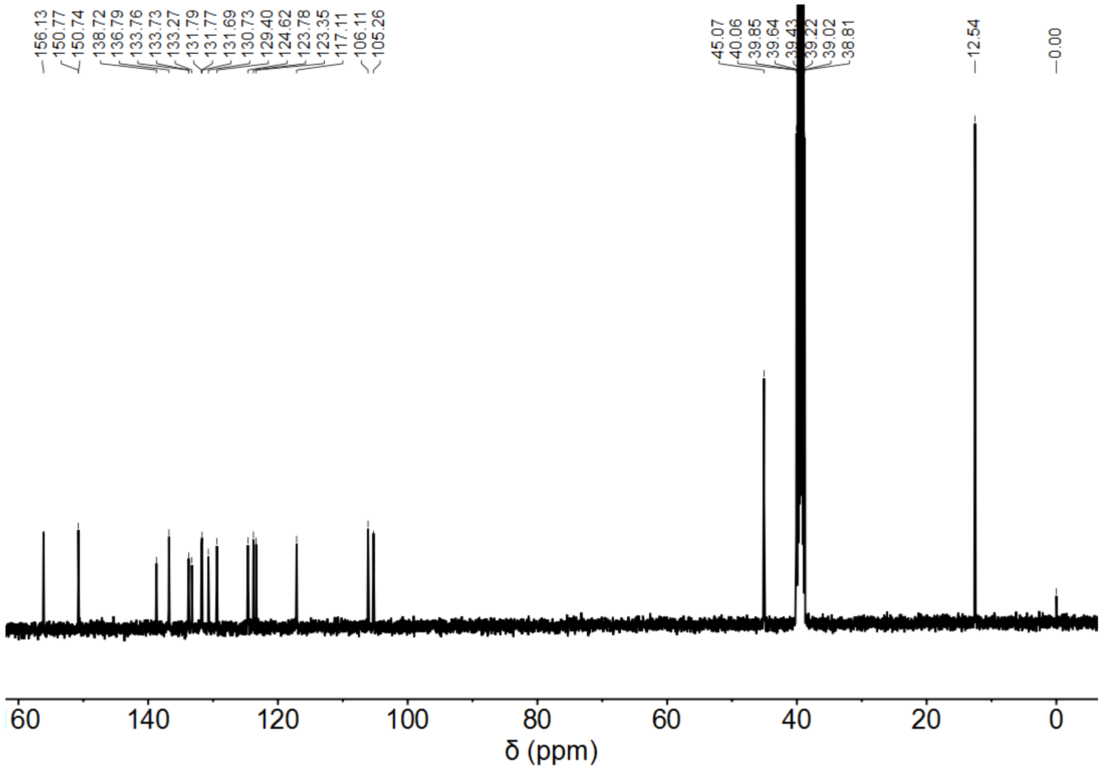

Figure S11. ^{13}CNMR of NH_2NBS in DMSO-d_6.

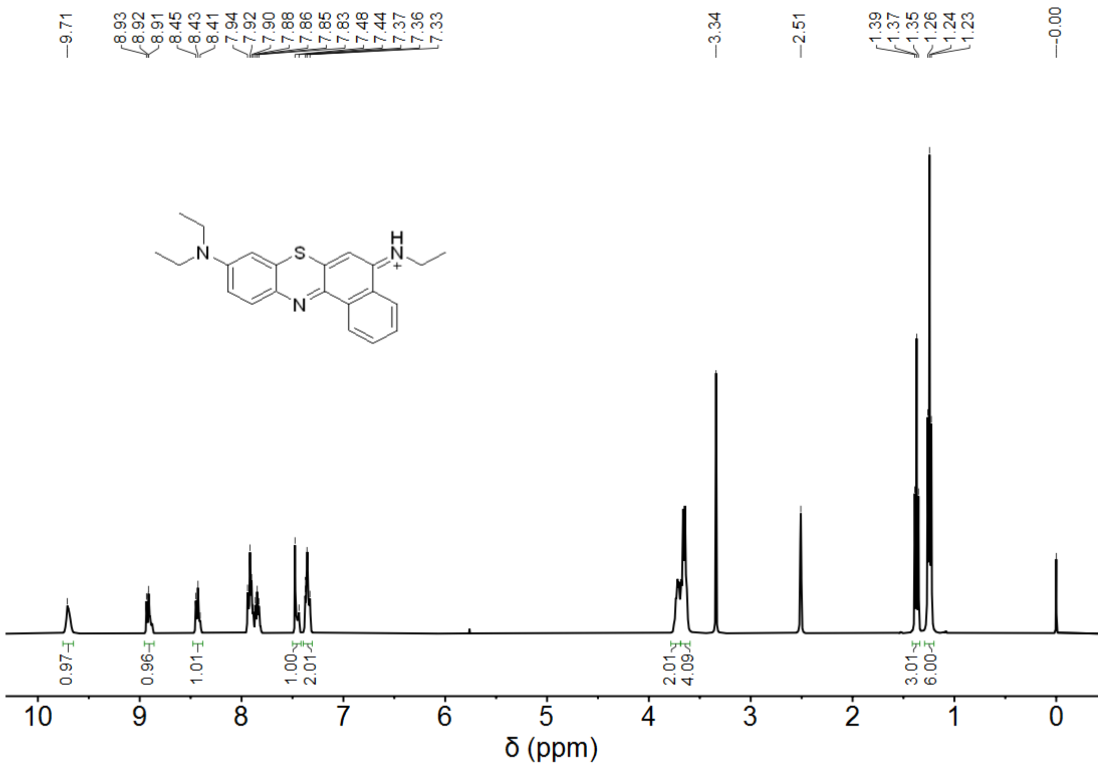

Figure S12. ^{1}HNMR of EtNBS in DMSO-d_6.

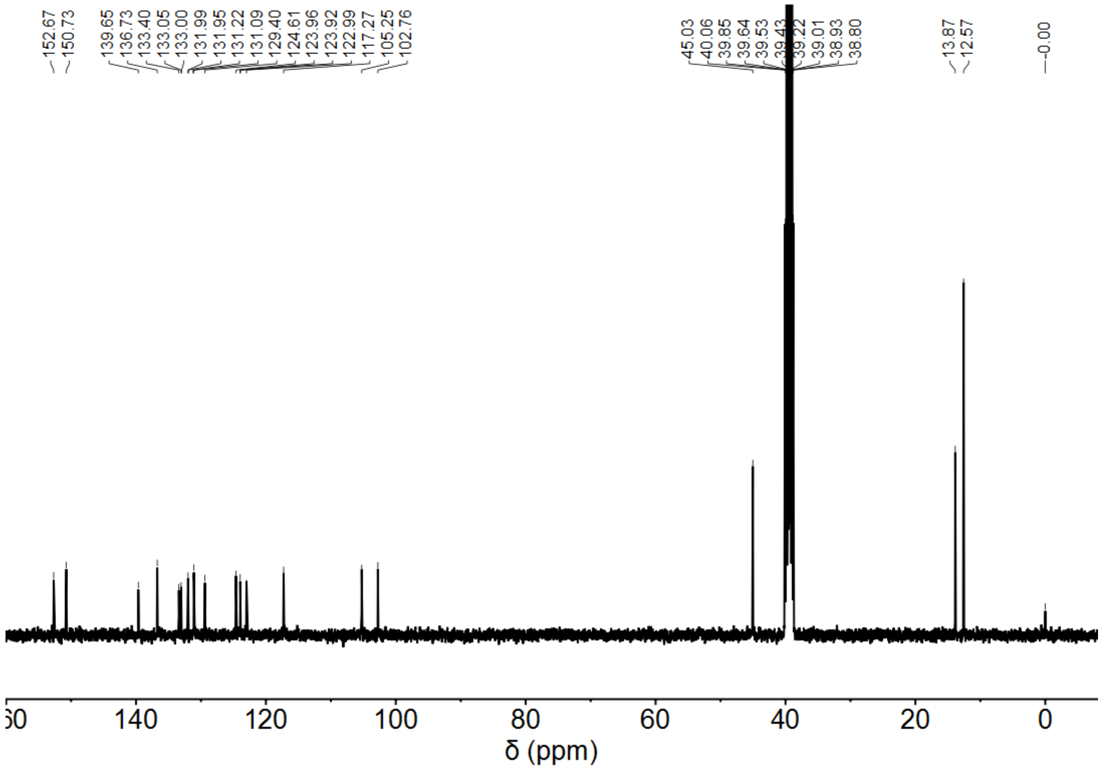

Figure S13. ^{13}CNMR of EtNBS in DMSO-d_6.

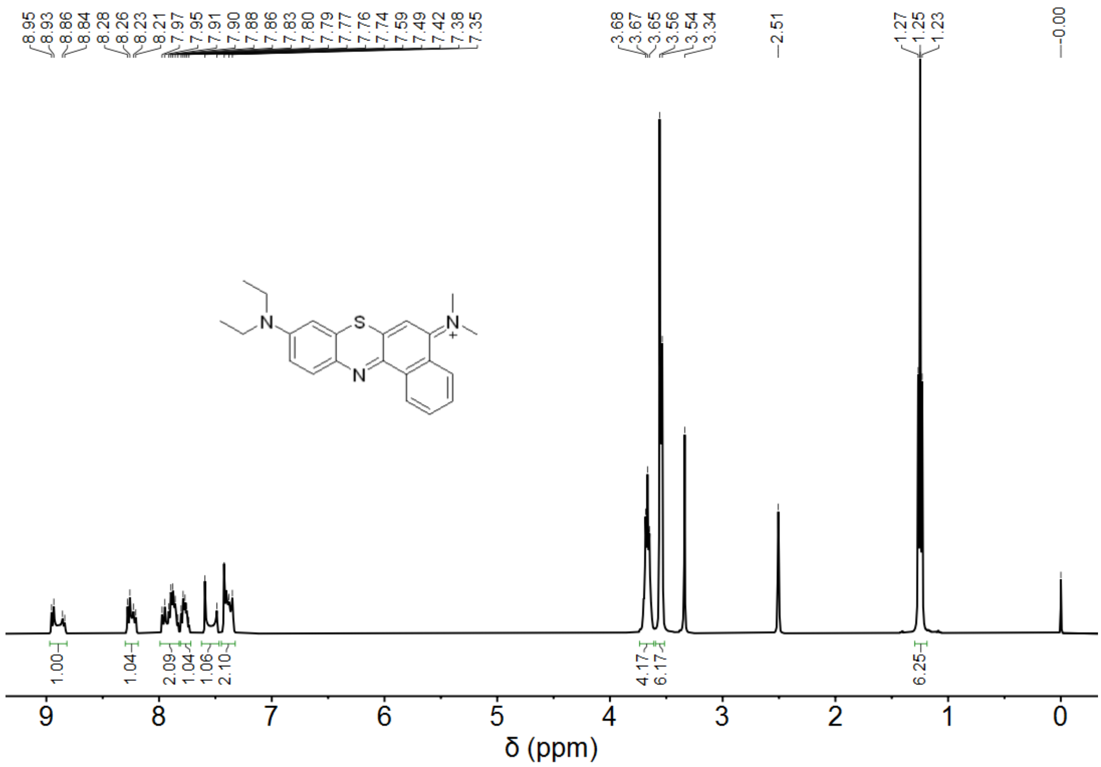

Figure S14. ^{1}HNMR of NMe_2NBS in DMSO-d_6.

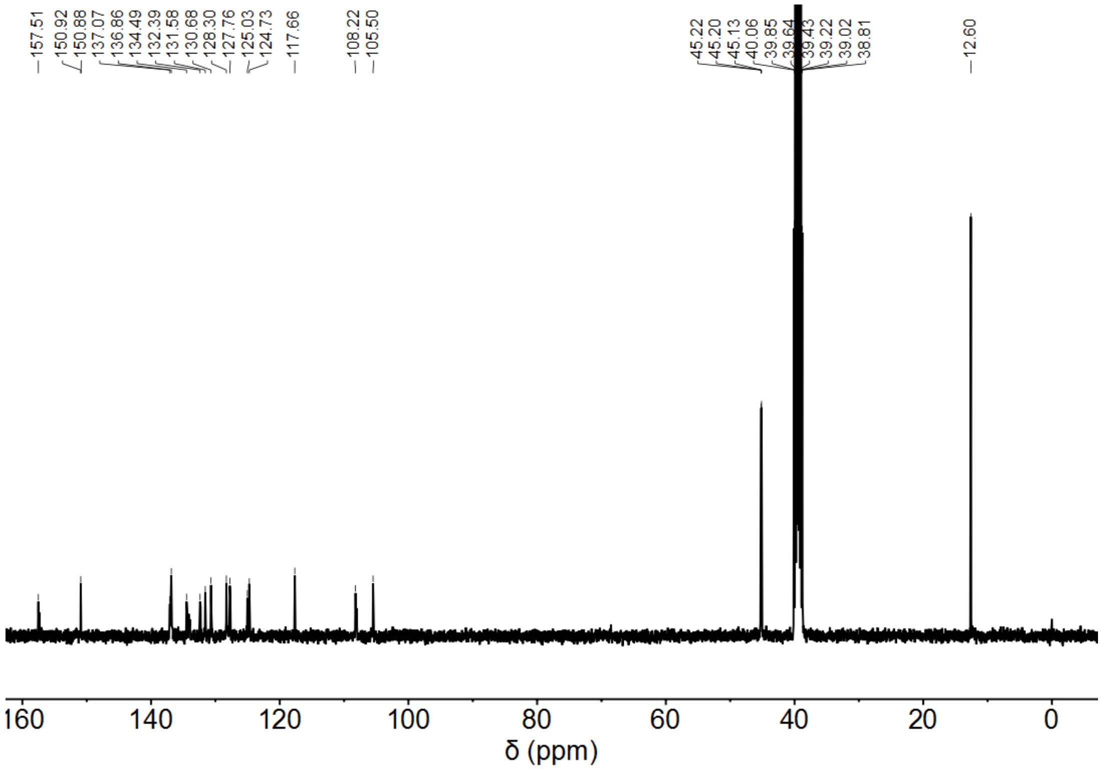

Figure S15. ^{13}CNMR of NMe_2NBS in DMSO-d_6.

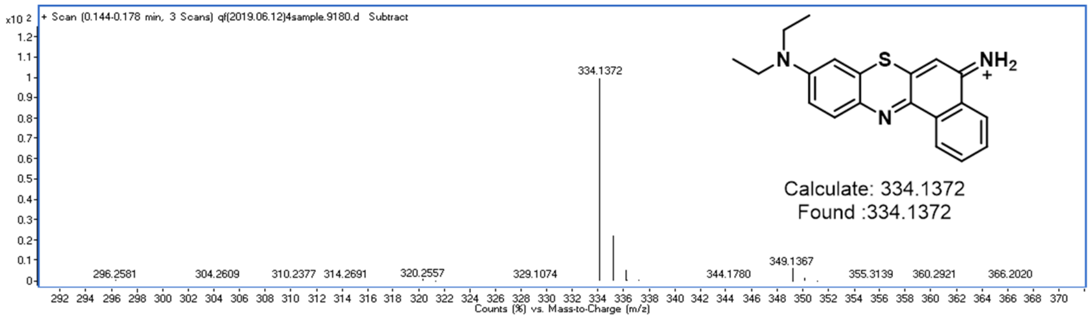

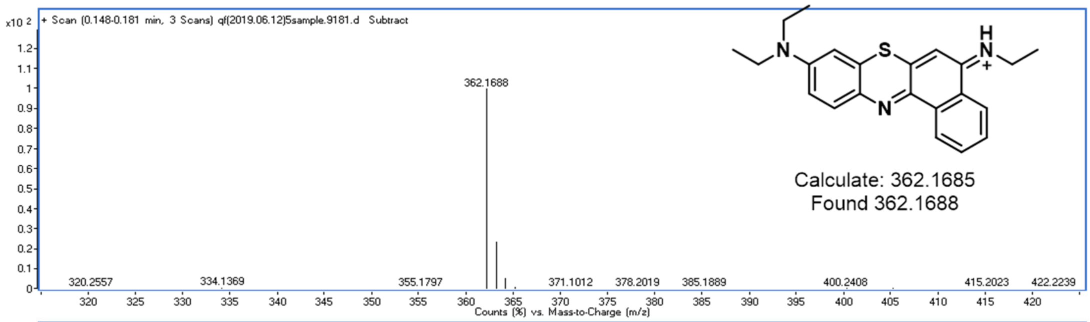

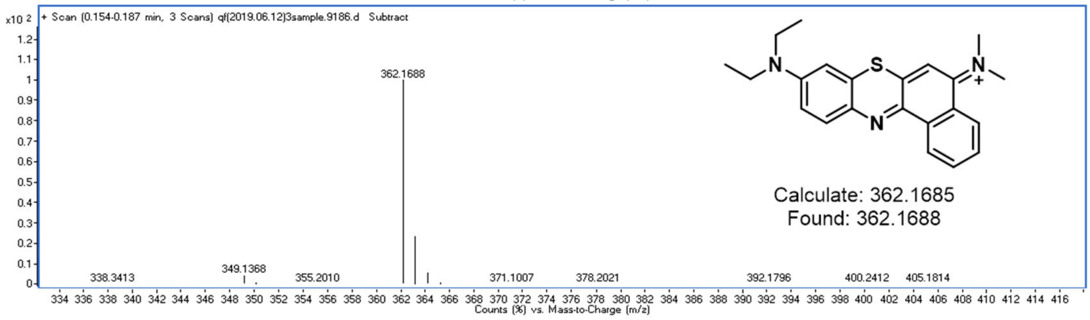

Figure S16. HRMS of NH_2NBS, EtNBS and NMe_2NBS.
